# Efficacy of N-Acetyl Cysteine in the Treatment of Burning Mouth Syndrome—A Randomized Controlled Trial

**DOI:** 10.3390/dj13080336

**Published:** 2025-07-23

**Authors:** Lorena Horvat Aleksijević, Božana Lončar Brzak, Miroslav Sikora, Ivana Škrinjar, Vlaho Brailo, Ana Andabak Rogulj, Marko Aleksijević, Danica Vidović Juras

**Affiliations:** 1 Health Centre Osijek—Baranja County, Park kralja P. Krešimira IV/6, 31000 Osijek, Croatia; lorenaa.bm@hotmail.com (L.H.A.); miro_jr@yahoo.com (M.S.); marko.aleksijevic@yahoo.com (M.A.); 2 Department of Oral Medicine, School of Dental Medicine, University of Zagreb, 10000 Zagreb, Croatiaanaandabak@gmail.com (A.A.R.); djuras@sfzg.unizg.hr (D.V.J.); 3 Department of Dental Medicine, Faculty of Dental Medicine and Health Osijek, J.J. Strossmayer University of Osijek, Crkvena 21, 31000 Osijek, Croatia; 4 Department of Oral Diseases, Dental Clinic, University Hospital Centre, Kišpatićeva 12, 10000 Zagreb, Croatia

**Keywords:** burning mouth syndrome, N-acetyl cysteine, placebo, neuropathic pain

## Abstract

**Objectives:** Burning mouth syndrome (BMS) is a chronic, painful, idiopathic condition of the oral cavity, characterized by the absence of visible pathological changes on the oral mucosa and normal laboratory findings. Recent evidence from the literature supports the classification of BMS as a neuropathic condition. It has been proposed that oxidative stress may contribute to neuropathic pain. N-acetylcysteine (NAC) is an antioxidant that exhibits neuroprotective properties. The aim of the study was to evaluate the efficacy of N-acetyl cysteine in the treatment of burning mouth syndrome (BMS). **Methods:** Eighty female patients with previously diagnosed BMS were randomly assigned to one out of two groups. One group received N-acetyl cysteine (600 mg/twice a day) and the other received placebo, for an eight-week period. The outcome was measured by the Oral Health Impact Profile-14 (OHIP-14) quality of life questionnaire and Numeric Pain Rating Scale, for burning and discomfort, both before and after completing the therapy. **Results:** Both groups experienced a significant reduction in burning and discomfort sensations, along with a significant improvement in oral health-related quality of life. However, the difference between the treatment and control group was not statistically significant. **Conclusions:** NAC does not significantly improve the oral health-related quality of life, burning sensations, and discomfort in BMS subjects compared to placebo.

## 1. Introduction

Burning mouth syndrome (BMS) is a chronic, painful, idiopathic condition of the oral cavity, characterized by the absence of visible pathological changes on the oral mucosa [1,2]. To confirm the diagnosis, a patient must experience burning sensations or altered oral sensations (dysesthesia) for more than two hours per day over a period of at least three months, without any observable clinical signs [3]. The reported prevalence of BMS in the general population varies, depending on diagnostic criteria, which were not clearly established in the past. According to the recent studies using current diagnostic criteria, the estimated prevalence in the general population is between less than 1% and 3.7% [4]. Most often it affects perimenopausal and postmenopausal women [1,2,3,4,5], with women being up to several times more likely to be affected than men [5].

The etiology of BMS remains unclear; however, it is considered to be multifactorial, likely resulting from a combination of neurophysiological and psychological disturbances. Due to its unclear etiology and the variety of proposed pathogenic mechanisms, currently, there is no definitive or universally accepted treatment for BMS. The primary goal of BMS treatment is to alleviate painful burning sensations and dysesthesia [5,6,7,8,9].

Increasing evidence, however, supports the classification of BMS as a neuropathic condition [1,2,5,10].

Neuropathic pain is triggered by lesions or diseases of the somatosensory nervous system, which alter its structure and function in such a way that pain arises spontaneously and is pathologically amplified in response to either harmful or non-harmful stimuli [11]. This type of pain may result from injury to either the central or peripheral nervous system [12].

BMS patients can be classified into two subgroups if it is assumed that BMS is a form of neuropathic pain. The first subgroup includes patients with subclinical peripheral neuropathic pain, caused by extensive but subclinical trigeminal neuropathy and trigeminal lesions or by small fiber neuropathy of the intraoral mucosa, accompanied by signs of functional loss that can be confirmed through quantitative sensory testing [13].

The second subgroup consists of patients with BMS who exhibit changes typical of central neuropathic pain, such as positive neurophysiological and neurotransmitter markers, indicating low levels of dopamine in the brain and an increased prevalence of psychiatric comorbidities associated with dopamine levels in BMS patients [4,5,14].

Recently, it has been proposed that oxidative stress may contribute to neuropathic pain. Oxidative stress refers to the increased production of free radicals or a decrease in antioxidant concentrations, leading to an imbalance between pro-oxidative and antioxidant molecules [15]. A growing body of research indicates that oxidative stress and neuroinflammation play a major role in the development and progression of diabetic neuropathy [16,17]. Pain is one of the most common symptoms and, when present, the condition is referred to as painful diabetic neuropathy [18,19,20]. It is characterized by tingling, burning, and stabbing sensations. The pain ranges from moderate to severe and significantly reduces quality of life [18,21,22]. The exact pathophysiological mechanisms of painful diabetic neuropathy are not yet fully understood, which makes its treatment particularly challenging. The prevailing view is that the condition is of multifactorial origin [18,23]. A clinical study conducted on patients with diabetic neuropathy demonstrated the efficacy of NAC (600 mg/twice a day, for 8 weeks) in treatment; however, no statistically significant difference was observed compared to the standard therapy with pregabalin [24]. Nevertheless, combined therapy with pregabalin and NAC proved to be significantly more effective than pregabalin combined with placebo [18].

Antioxidants are molecules that counteract free oxygen radicals and their harmful effects, thereby reducing oxidative stress and the diseases associated with it. Lowering the concentration of free radicals helps preserve normal cellular functions [25]. N-acetylcysteine (NAC) is an antioxidant that exhibits neuroprotective properties, which is why it is used in the treatment of neurodegenerative and psychiatric disorders [26,27]. Alzheimer’s disease is the most common type of dementia and is defined as a slowly progressive neurodegenerative disorder [28]. Increasing evidence links the onset of Alzheimer’s disease to an imbalance between oxidants and antioxidants. A shift toward oxidative processes is thought to contribute to the formation of amyloid plaques and the accumulation of β-amyloid and neurofibrillary tangles. However, it remains unclear whether oxidative stress initiates their formation or whether their presence induces oxidative stress [29]. NAC has been the subject of numerous studies investigating the role of antioxidants in Alzheimer’s disease. A nutraceutical formulation containing folic acid, vitamin B12, alpha-tocopherol, acetyl-L-carnitine, and NAC demonstrated greater efficacy than placebo in patients with Alzheimer’s disease [30,31].

Parkinson’s disease is a complex, progressive neurodegenerative disorder and ranks as the second most common neurodegenerative disease after Alzheimer’s disease. One of the potential contributing factors to the pathogenesis of Parkinson’s disease is oxidative stress and the resulting mitochondrial dysfunction, which play a role in the cascade leading to the degeneration of dopaminergic neurons [32]. For this reason, antioxidants, including NAC, are being considered as potential therapeutic options in the management of Parkinson’s disease. Clinical studies have demonstrated significant increases in dopamine transporter binding following NAC administration [33,34].

Psychiatric disorders have a multifactorial etiology that includes inflammatory processes, glutamate transport, oxidative stress, mitochondrial function, apoptosis, dopamine pathways, etc. [35]. Given that N-acetyl cysteine participates in most of these pathways, a large number of clinical studies have been published that have monitored the effect of NAC as an adjunctive treatment for a number of different psychiatric disorders (depression, bipolar disorder, schizophrenia, obsessive–compulsive disorder, various forms of addiction, pathological gambling, etc.) [36,37,38]. In most cases, NAC has had a positive effect in clinical applications.

As a result, the present study wanted to determine whether N-acetylcysteine might be useful in the treatment of burning mouth syndrome.

## 2. Materials and Methods

This study was approved by the Ethical Committee of the School of Dentistry, University of Zagreb, Croatia and Ethical Committee of Health Center of Osijek-Baranja County. The research was part of a doctoral dissertation study, approved by the Ethics Committee, School of Dentistry, University of Zagreb, Croatia (approval number: 05-PA-30-III-12/2021) and the institutional review board of Health Centre Osijek—Baranja County (approval number: 03-747-2/23). The research was registered at the U.S. National Institutes of Health (clinicaltrials.gov) (trial identifier: NCT05309070). Each patient signed informed consent according to the Declaration of Helsinki. All participants were informed about the study and provided with an information leaflet. All participants also signed informed consent. At the beginning of the study, participants completed the Oral Health Impact Profile questionnaire (OHIP-14) to assess self-perceived quality of life and rated the severity of their symptoms using the Numeric Pain Rating Scale (NPRS), ranging from 0 (no symptoms) to 10 (most severe symptoms) for burning symptoms and for discomfort from burning symptoms. These assessments were repeated at a follow-up visit two months after treatment was completed. Participants were randomly assigned to one of two groups, N-acetyl cysteine or placebo group. Both participants and the care providers were blinded to group allocation.

### 2.1. Eligibility Criteria

The participants in this study were patients diagnosed with BMS, according to the World Health Organization criteria, who met the inclusion criteria and did not exhibit any exclusion criteria. They were recruited from two locations: the Department of Oral Medicine at the School of Dentistry, University of Zagreb, and from the specialist oral medicine practice at the Health Center of Osijek-Baranja County. After the study was explained to them, each participant provided a detailed medical history, underwent both extraoral and intraoral clinical examinations, and gave written informed consent. The inclusion criteria were adult patients over the age of 18, the presence of burning or stinging sensations in the oral mucosa persisting for more than three months, and clinically normal oral mucosa without pathological changes. The exclusion criteria were iron deficiency, vitamin B deficiency, pregnant or breastfeeding women, women planning pregnancy, patients with active gastric or duodenal ulcers, and patients in which diagnosis of BMS was just established. Newly diagnosed patients with BMS were excluded to eliminate the placebo effect of any treatment, because some patients feel relief after only establishing a diagnosis and receiving a detailed explanation and instructions, and they do not seek further help.

### 2.2. Study Setting

The study was conducted at two locations in Croatia: the Department of Oral Medicine at the School of Dental Medicine, University of Zagreb, and a specialist oral medicine practice at the Health Center of Osijek-Baranja County. Both sites provided outpatient care to patients with various oral mucosal conditions and served as recruitment centers for the study.

### 2.3. Interventions

A total of 80 patients were included in the study; 40 of them were randomly assigned to the treatment group and 40 to the control group. N-acetylcysteine and placebo were packaged in identical, unmarked, numbered white opaque bottles containing white opaque capsules to ensure visual indistinguishability. The numerical code was revealed at the end of the study, prior to statistical analysis of the results.

The treatment group consisted of 40 randomly selected patients who received N-acetylcysteine at a total daily dose of 1200 milligrams (mg), administered as two 600 mg capsules taken twice daily, in the morning with breakfast and in the evening after dinner, for two months. The control group included 40 patients who received placebo capsules containing starch which were taken the same way. To make the groups more homogeneous, only female patients were included in the study.

### 2.4. Outcome Measures

At both the initial and follow-up visits, all participants completed the Croatian version of the OHIP-14 questionnaire, which measures oral health-related quality of life, and rated the intensity of their symptoms using the Numeric Pain Rating Scale (NPRS), specifically for burning sensations and discomfort. The primary outcome measure was improvement in patient’s quality of life as determined by a self-perceived quality of life questionnaire (OHIP-14). Quality of life was established based on the sum of participants’ answers to the 14 questions. The answer options with their respective values were as follows: 0 = never, 1 = rarely, 2 = sometimes, 3 = repeatedly, 4 = always, with a maximum score of 56. The higher the score, the worse the quality of life. OHIP-14 was filled out after inclusion in the study (before treatment) and at the control examination after two months of treatment.

Secondary outcome measures were improvement in patient’s subjective burning symptoms and discomfort as measured on a numeric pain rating scale (NPRS) grading from 0 to 10 (0 = without symptoms, 10 = worst possible symptoms) [31]. Burning symptoms and discomfort were determined on the NPRS after inclusion in the study (before treatment) and at the control examination after two months of treatment.

### 2.5. Sample Size Determination

The sample size was determined using MedCalc version 23 statistical software, based on a power analysis with a statistical power of 80% and a significance level of α = 0.05. The calculation was guided from the literature data [39], assuming a positive response rate of 64% in the treatment group and 28% in the placebo group. A minimum of 28 participants per group was required to achieve the desired statistical power. To account for potential dropouts, the number of participants in each group was increased to 40.

### 2.6. Allocation

Patients were randomly assigned to one of two groups using a randomization sequence generated by the random number generator and subsequently allocated to their respective interventions.

### 2.7. Blinding

After allocation into one of two groups, both the participants and the care providers were blinded.

### 2.8. Statistical Analysis

The data were analyzed using the MedCalc23 statistical software. The difference in NPRS and OHIP-14 values before and after therapy was tested using the Wilcoxon signed-rank test for two related samples. The reduction in NPRS and OHIP-14 scores between groups was calculated using the Mann–Whitney test. A significance level of 0.05 (*p* < 0.05) was chosen for all tests. Standardized effect size was calculated for OHIP-1 and OHIP-2 in patients with different types of therapy.

## 3. Results

**Figure 1:**
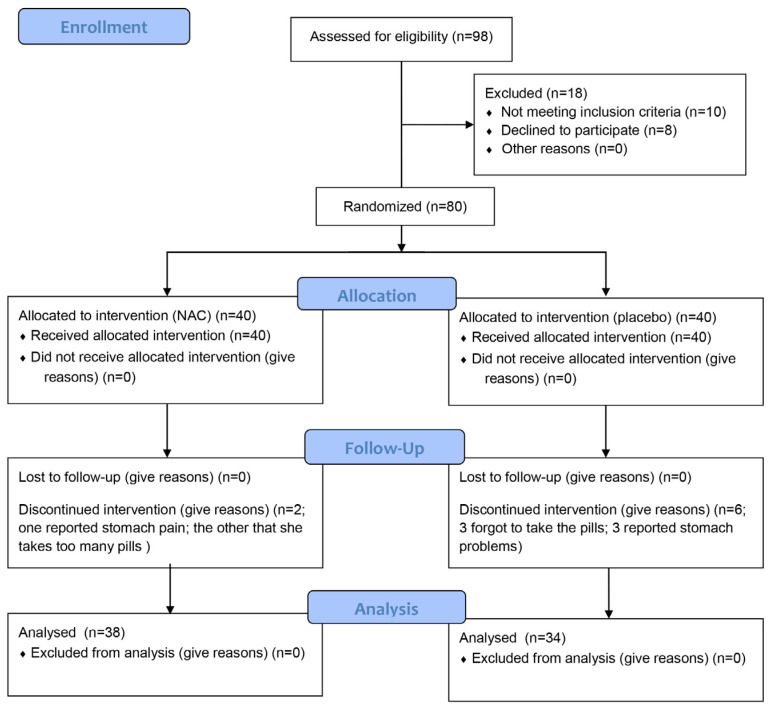
Flow chart of the participant enrollment.

A total of 80 participants were enrolled in this study and randomly assigned to two groups of equal size. All participants met the inclusion criteria; however, eight participants withdrew before the study was completed. Six participants from the control group and two from the treatment group discontinued their participation (Figure 1). The reported reasons for withdrawal included stomach pain (one patient in the treatment group, three in control group), forgetfulness in taking the capsules (three patients in control group), or the fact that the patient “already takes a lot of pills” (one patient in the treatment group). The treatment and control groups were well matched in terms of age, with a median age of 69 years in the treatment group and 74 years in the control group (Table 1). No significant difference in age was found between test and control group (*p* = 0.107, Kruskal–Wallis test).

Statistical analysis demonstrated significantly lower OHIP-14 questionnaire scores in both groups. A greater improvement in oral health-related quality of life was observed in the placebo group (median score reduced from 24 to 11), compared to the treatment group (median score reduced from 20.5 to 13) (Table 2).

NPRS scores for burning sensation were significantly lower in both groups after the intervention (Wilcoxon signed-rank test, *p* < 0.001). In Group 1, the median burning score decreased from 6 at the first visit to 4 at the second visit. Similarly, in Group 2, the median score decreased from 7 to 4 (Table 3).

NPRS scores for discomfort were also significantly reduced in both groups following the intervention (Wilcoxon signed-rank test, *p* < 0.001). In Group 1, the median discomfort score dropped from 7 at baseline to 4 at follow-up, while in Group 2, the score decreased from 7 to 4, representing a reduction of approximately 2.5 points (Table 4).

## 4. Discussion

Burning mouth syndrome is a chronic painful condition of the oral cavity that predominantly affects the mucosa of the tongue and primarily occurs in perimenopausal and postmenopausal women [40,41]. Due to its complex etiology and unclear pathogenesis, there is still no standardized treatment protocol suitable for all patients, making the management of BMS a significant challenge for clinicians [40]. Recent theories suggest neurological, endocrinological, and psychological factors as potential causes of BMS, although the most widely accepted theory currently describes BMS as a neuropathic pain. According to current IHS classification [3], the existence of a secondary burning mouth syndrome which would be attributed to a local or systemic factor is questionable. Patients with BMS frequently suffer from stress-related disorders and experience a reduced quality of life [41,42,43,44,45]. Most treatment approaches for BMS focus on alleviating clinical symptoms—relieving the burning sensation and oral pain, promoting nerve fiber regeneration, correcting nutritional deficiencies and hormonal imbalances, and providing psychological support and care [45].

Reactive oxygen species (ROS), under normal physiological conditions, play a crucial role in various cellular processes such as differentiation, proliferation, growth, and apoptosis. However, when present in excess, ROS can cause damage to cellular structures, including lipids, proteins, and nucleic acids, leading to a condition known as oxidative stress. Oxidative stress refers to an elevated level of reactive oxygen species, and it is now believed that many chronic pain conditions, including neuropathic pain, are associated with oxidative stress [46,47]. The literature supports the correlation between oxidative stress and BMS, although it still does not definitively prove causation. It is not clear whether oxidative stress causes BMS or is a consequence of it. Some studies have found biomarkers of oxidative stress to be elevated in patients with BMS compared to healthy controls, such as reactive oxygen species and biological antioxidant potential as iron-reducing activity, suggesting oxidative damage in the oral mucosa [48], or decreased levels of salivary FRAP (ferric reducing ability of plasma) and uric acid, suggesting the presence of oxidative stress [49].

An incomplete understanding of the pathophysiological mechanisms underlying neuropathic pain has prompted research into potential new therapeutic agents aimed at alleviating its symptoms. One such hypothesis suggests that nociceptive transmission may be inhibited by NAC, due to its known antioxidant properties [50]. Following oral administration, NAC is metabolized into glutathione, a potent antioxidant whose deficiency is believed to contribute to the development of peripheral neuropathy [51,52,53,54,55]. Glutathione exhibits strong antioxidant and anti-inflammatory effects, helping to reduce oxidative stress—a factor implicated not only in the onset but also in the progression of chronic pain and inflammatory conditions [46].

Horst et al. observed that NAC induces a reduction in nitric oxide metabolites and enhances the activity of antioxidant enzymes such as glutathione peroxidase (GPx) and glutathione S-transferase in an experimental rat model [51]. Administration of NAC may offer protection against the early rise in lipid hydroperoxide levels by increasing the concentration of ascorbic acid and mitigating the decline in total antioxidant capacity observed in later stages [56]. The protein P-p38, identified as a mediator of neuropathic pain, showed decreased expression following NAC treatment in rats, which also led to improved recovery of the sciatic nerve [57]. Pain threshold is influenced by group II metabotropic glutamate receptors and the xc– system, which mediates the release of intracellular glutamate [56,57,58,59,60,61]. NAC has been shown to induce analgesia by enhancing the activity of both the xc–system and type II metabotropic glutamate receptors in models of acute and chronic pain [62]. Furthermore, NAC affects neuropathic pain by inhibiting matrix metalloproteinases (MMPs) and blocking the release of interleukin-1β (IL-1β), a critical substrate of MMPs [63]. Harmful oxygen radicals can activate transient receptor potential (TRP) cation channels in dorsal root ganglion neurons, contributing to neuropathic pain; NAC is capable of modulating these cation channels, thereby offering protection against oxidative stress. NAC administration has also been shown to reduce levels of lipid peroxidation in dorsal root ganglion neurons [64].

The study conducted by Han et al. on patients suffering from BMS demonstrated the efficacy of NAC as a monotherapy (400 mg a day) as well as in combination therapy with clonazepam for the treatment of BMS. The control group received clonazepam alone, a previously established effective treatment; however, a limitation of the study was the absence of a placebo-controlled group. The combination therapy yielded superior outcomes compared to both monotherapies, with results evaluated using the OHIP-14 questionnaire and the Visual Analog Scale (VAS) [65]. Recent results have shown that clonazepam was efficient in treating burning mouth syndrome only in patients with concomitant taste disturbances, but not in all BMS patients [66]. To date, our study and the study of Han et al. are the only two investigations that have specifically focused on the efficacy of NAC in the treatment of BMS. Nonetheless, NAC has previously shown therapeutic potential in other neuropathic conditions and neurodegenerative diseases.

N-acetylcysteine is not the only antioxidant that has attracted interest among researchers as a potential therapy for BMS. Alpha-lipoic acid (ALA) is a potent antioxidant naturally produced in the body and also found in various foods. It has long been used as a treatment for diabetic neuropathy and as an adjuvant therapy in managing radiotherapy-related complications [67]. Palacios-Sánchez et al. demonstrated the efficacy of ALA in patients with BMS through a double-blind randomized controlled trial comparing ALA to placebo. In the treatment group, 64% of patients reported symptom improvement, with 68.75% maintaining results one month post-treatment [39]. Similarly, Femiano and Scully reported effectiveness of ALA in 97% of patients, with 73% confirming sustained improvement one year after therapy [68]. In another study, López-D’Alessandro and Escovich found ALA monotherapy effective in 55% of BMS patients. However, the group receiving combined therapy with ALA and gabapentin achieved markedly superior results, with 70% of participants reporting symptom reduction [69]. A recently published systematic review about this topic has concluded that the efficiency of ALA in treatment of BMS still needs to be evaluated [70].

The placebo effect is a complex psychosocial phenomenon with a complex neurobiological basis which is associated with different diseases and therapeutic modalities. Evidence from different methodological approaches have shown that the placebo effect is real and that placebo analgesia is regulated by the endogeneous opioid mechanism [71,72]. The results of the present study have shown a significant reduction in symptoms and a significant improvement in OHRQL in both groups of patients. The placebo effect depends on numerous factors, from the psychosocial context in which a therapy is applied, to the characteristics of the person themselves (positive expectation, belief). Results from the literature show that a positive treatment response to placebo is frequent in studies evaluating treatment options in BMS [73]. These results also suggest that psychological factors related to BMS must be considered.

A review by Kuten-Shorer et al. [73] included 12 randomized clinical trials that evaluated the effectiveness of the treatment of burning mouth syndrome. Of those 12, 10 studies recorded a positive response to treatment, of which a positive response to placebo was also observed in 6 studies (6/10, 60%). The positive response to placebo varied, according to their data, from 15 to 74%. The average placebo response as a proportion of drug response across all ten studies was 72%, indicating a strong placebo response. In as many as three studies, the placebo was as effective as the active drug. Cavalvanti and Silveira [74] evaluated the efficacy of systemic alpha-lipoic acid (ALA) compared to cellulose starch. Carbone et al. [75] examined the efficacy of systemic ALA, ALA combined with multivitamins, and a placebo containing cellulose. Both studies showed statistically significant improvement in symptoms in all three groups, with no difference in the efficacy of the active drug and placebo. The third study evaluated the efficacy of the antidepressant trazodone for the treatment of burning mouth syndrome, compared to placebo [76]. The placebo group showed a statistically significant improvement that did not differ from the group receiving the active substance.

The authors of the previously mentioned review paper also stated that not all published papers state the composition of the placebo and that the placebo formulations used are not unique either [73]. Starch, cellulose, or lactose monohydrate are listed as the main ingredients, and boric acid was used as a placebo solution for the oral cavity. Also, with some authors, there is no data on the durability of the results. The authors recommended that future studies include a period of monitoring the durability of the results lasting at least two months. Given the high efficacy of placebo in previous studies of BMS treatment and the limitations of subjective outcome assessment through patient self-report in the case of BMS, they also suggested that future studies include a group of patients who will not receive therapy at all [73,77]. In this way, it would be possible to distinguish whether there is an improvement in symptoms due to the course of the disease itself and a true placebo effect. Our research did not include a group that did not receive any treatment, but all patients were previously diagnosed and thoroughly informed about the condition, after which they have not received other treatment. Some patients feel a certain relief of complaints after being informed about the condition and do not seek other types of treatment. In this way, we wanted to rule out the placebo effect of the information about the condition itself and the conversation with the patient on the intensity of complaints, which is in line with this recommendation.

## 5. Conclusions

The present clinical study demonstrated that N-acetyl cysteine therapy is not more effective in alleviating burning and discomfort or improving quality of life in patients suffering from BMS compared to placebo. Both patient groups have reported significant improvement of all parameters after treatment. Nevertheless, the results need to be confirmed by further studies with a greater number of participants. Some studies have shown promising results when NAC is combined with other medications. Future research should also evaluate the effectiveness of combining NAC with other medications, such as pregabalin, in treating burning mouth syndrome.

## Figures and Tables

**Table 1:** Age distribution among the therapeutic and placebo group (*p* > 0.05, Kruskal–Wallis test).

	Age (Median, Range) (Years)	*p*
Group 1	69 (44–93)	0.107
Group 2	74 (38–96)	

**Table 2:** Differences between OHIP-1 and OHIP-2 scores in BMS patients in the therapeutic and placebo group (Wilcoxon signed-rank test).

	Median (Range)		Difference	95% Confidence Interval	*p* *	Standardized Effect Size
	OHIP-1 *	OHIP-2 **				
OHIP-14						
Group 1 (38)	20.5 (14–26)	13 (9–19)	−6	−8 to −4	<0.001	0.7
Group 2 (34)	24 (21–33)	11 (7–20)	−10.5	−14.5 to −6.5	<0.001	0.9

* measured before the beginning of the treatment; ** measured after two months of treatment.

**Table 3:** Differences between NPRS-1 and NPRS-2 burning scores in BMS patients in therapeutic and placebo group (Wilcoxon signed-rank test).

	Median (Range)		Difference	95% Confidence Interval	*p* *
	NPRS-1 *	NPRS-2 **			
NPRS—burning					
Group 1 (38)	6 (5–8)	4 (3–5)	−2	−2.5 to −1.5	<0.001
Group 2 (34)	7 (5–8)	4 (3–5)	−2	−3 to −1.5	<0.001

* measured before the beginning of the treatment; ** measured after two months of treatment.

**Table 4:** Differences between NPRS-1 and NPRS-2 discomfort scores in BMS patients in therapeutic and placebo group (Wilcoxon signed-rank test).

	Median (Range)		Difference	95% Confidence Interval	*p* *
	NPRS-1 *	NPRS-2 **			
NPRS—discomfort					
Group 1 (38)	7 (5–8)	4 (3–6)	−2.5	−3 to −1.5	<0.001
Group 2 (34)	7 (6–8)	4 (2–5)	−2.5	−3.5 to −1.5	<0.001

* measured before the beginning of the treatment; ** measured after two months of treatment.

## Data Availability

The raw data supporting the conclusions of this article will be made available by the corresponding author on request.
